# Supplementary material for: Cellular and Molecular Targets for Non-Invasive, Non-Pharmacological Therapeutic/Rehabilitative Interventions in Acute Ischemic Stroke
Source: Int J Mol Sci. 2022 Jan 14;23(2):907. doi: 10.3390/ijms23020907 (PMC8846361; doi:10.3390/ijms23020907)
Supplement: Supplementary file 1 [file ijms-23-00907-s001.zip › ijms-1536760-supplementary.pdf]

Cellular and Molecular Targets for Non-invasive, Non-pharmacological therapeutic/rehabilitative interventions in Acute Ischemic Stroke – Systematic Review

Table S1. The 27 sets of keywords / combinations of keywords/syntaxes used for the contextual searches in 4 international databases

Keywords	Elsevier	PubMed	PMC	PEDro	Total
"Acute Ischemic Stroke" + "Neuroprotection" + "Neurologic Music Therapy"	0	0	0	0	0
"Acute Ischemic Stroke" + "Neuroprotection" + "Photobiomodulation"	0	0	19	0	19
"Acute Ischemic Stroke" + "Neuroprotection" + "Transcranial Direct Current Stimulations"	0	0	0	0	0
"Acute Ischemic Stroke" + "Neuroprotection" + "TDCS"	0	0	19	0	19
"Acute Ischemic Stroke" + "Neuroprotection" + "Electro-Acupuncture"	0	0	27	0	27
"Acute Ischemic Stroke" + "Neuroprotection" + "Hyperbaric oxygen environment"	0	0	0	0	0
"Acute Ischemic Stroke" + "Neuroprotection" + "CO2 therapy"	0	0	0	0	0
"Acute Ischemic Stroke" + "Neuroprotection" + "Hyperbaric oxygen therapy"	0	2	35	0	37
"Acute Ischemic Stroke" + "Neuroprotection" + "HBOT"	0	2	9	0	11
"Acute Ischemic Stroke" + "Neuroprotection" + "Physiatric Intervention"	0	0	0	0	0
"Acute Ischemic Stroke" + "Neuroprotection" + "Physiatric Interventions"	0	0	0	0	0
"Acute Ischemic Stroke" + "Neuroprotection" + "Rehabilitative Intervention"	0	0	2	0	2
"Acute Ischemic Stroke" + "Neuroprotection" + "Rehabilitative Interventions"	0	0	0	0	0
"Acute Ischemic Stroke" + "Neuroprotection" + "Transcranial Magnetic Stimulation"	0	0	41	0	41
"Acute Ischemic Stroke" + "Neuroprotection" + "TMS"	0	0	28	0	28
"Acute Ischemic Stroke" + "Neuroprotection" + "Repetitive Transcranial Magnetic Stimulation"	0	0	29	0	29
"Acute Ischemic Stroke" + "Neuroprotection" + "RTMS"	0	0	18	0	18
"Acute Ischemic Stroke" + "Neuroprotection" + "Systemic Cooling"	0	3	18	0	21
"Acute Ischemic Stroke" + "Neuroprotection" + "Selective Brain Cooling"	0	0	25	0	25
"Acute Ischemic Stroke" + "Neuroprotection" + "Targeted Brain Cooling"	0	0	4	0	4
"Acute Ischemic Stroke" + "Neuroprotection" + "Selective Cerebral Cooling"	0	0	0	0	0
"Acute Ischemic Stroke" + "Neuroprotection" + "Targeted Brain Cooling"	0	0	4	0	4
"Acute Ischemic Stroke" + "Neuroprotection" + "Systemic Therapeutic Hypothermia"	0	0	3	0	3
"Acute Ischemic Stroke" + "Neuroprotection" + "Selective Brain Hypothermia"	0	2	13	0	15
"Acute Ischemic Stroke" + "Neuroprotection" + "Targeted Brain Hypothermia"	0	0	0	0	0
"Acute Ischemic Stroke" + "Neuroprotection" + "Selective Cerebral Hypothermia"	0	0	10	0	10
"Acute Ischemic Stroke" + "Neuroprotection" + "Targeted Brain Hypothermia"	0	0	0	0	0
Total	**0**	**9**	**304**	**0**	**313**

Table S2. The articles that satisfied all the previous filtering criteria / PRISMA stages selected for qualitative synthesis without meta-analysis

Authors	Title	Publishing details	Ref. no.	Keywords	Year	Score
"Rafał Szelenberger, Joanna Kostka, Joanna Saluk-Bijak, Elżbieta Miller"	"Pharmacological Interventions and Rehabilitation Approach for Enhancing Brain Self-repair and Stroke Recovery"	"Curr Neuropharmacol. 2020 Jan; 18(1): 51–64. Published online 2020 Jan. doi: 10.2174/1570159X17666190726104139"	(63)	"Acute Ischemic Stroke" + "Neuroprotection" + "TDCS"	2020	10
"Ciprian Hentia, Alex Rizzato, Enrico Camporesi, Zhongjin Yang, Danina M. Muntean, Dorel Săndesc, Gerardo Bosco"	"An overview of protective strategies against ischemia/reperfusion injury: The role of hyperbaric oxygen preconditioning"	"Brain Behav. 2018 May; 8(5): e00959. Published online 2018 Mar 30. doi: 10.1002/brb3.959"	(7)	"Acute Ischemic Stroke" + "Neuroprotection" + "Hyperbaric oxygen therapy"	2018	10
"Qwang-Yuen Chang, Yi-Wen Lin, Ching-Liang Hsieh"	"Acupuncture and neuroregeneration in ischemic stroke"	"Neural Regen Res. 2018 Apr; 13(4): 573–583. doi: 10.4103/1673-5374.230272"	(82)	"Acute Ischemic Stroke" + "Neuroprotection" + "Electro-Acupuncture"	2018	10
"Yiming Deng, Duanduan Chen, Feng Gao, Hong Lv, Guojun Zhang, Xuan Sun, Lian Liu, Dapeng Mo, Ning Ma, Ligang Song, Xiaochuan Huo, Tianyi "Yan, Jingbo Zhang, Zhongrong Miao"	"Exosomes derived from microRNA-138-5p-overexpressing bone marrow-derived mesenchymal stem cells confer neuroprotection to astrocytes following ischemic stroke via inhibition of LCN2"	"J Biol Eng. 2019; 13: 71. Published online 2019 Aug 28. doi: 10.1186/s13036-019-0193-0"	(26)	"Acute Ischemic Stroke" + "Neuroprotection" + "Photobiomodulation"	2019	8
"Yongxing Lai, Peiqiang Lin, Manli Chen, Yixian Zhang, Jianhao Chen, Mouwei Zheng, Ji Liu, Houwei Du, Ronghua Chen, Xiaodong Pan, Nan Liu, Hongbin Chen"	"Restoration of L-OPA1 alleviates acute ischemic stroke injury in rats via inhibiting neuronal apoptosis and preserving mitochondrial function"	"Redox Biol. 2020 Jul; 34: 101503. Published online 2020 Mar 13. doi: 10.1016/j.redox.2020.101503"	(33)	"Acute Ischemic Stroke" + "Neuroprotection" + "Electro-Acupuncture"	2020	8
"Yi Zhang, Ying Zhang, Xiao-fei Jin, Xiao-hong Zhou, Xian-hui Dong, Wen-tao Yu, Wei-juan Gao"	"The Role of Astragaloside IV against Cerebral Ischemia/Reperfusion Injury: Suppression of Apoptosis via Promotion of P62-LC3-Autophagy"	"Molecules. 2019 May; 24(9): 1838. Published online 2019 May 13. doi: 10.3390/molecules24091838"	(45)	"Acute Ischemic Stroke" + "Neuroprotection" + "Electro-Acupuncture"	2019	8
"Amir Hadanny, Mor Rittblat, Mor Bitterman, Ido May-Raz, Gil Suzin, Rahav Boussi-Gross, Yonatan Zemel, Yair Bechor, Merav Catalogna, Shai Efrati"	"Hyperbaric oxygen therapy improves neurocognitive functions of post-stroke patients – a retrospective analysis"	"Restor Neurol Neurosci. 2020; 38(1): 93–107. Prepublished online 2020 Jan 21. Published online 2020 Feb 11. doi: 10.3233/RNN-190959"	(91)	"Acute Ischemic Stroke" + "Neuroprotection" + "Hyperbaric oxygen therapy"	2020	8
"S. T. Peruzzaro, M. M. M. Andrews, A. Al-Gharaibeh, O. Pupiec, M. Resk, D. Story, P. Maiti, J. Rossignol, G. L. Dunbar"	"Transplantation of mesenchymal stem cells genetically engineered to overexpress interleukin-10 promotes alternative inflammatory response in rat model of traumatic brain injury"	"J Neuroinflammation. 2019; 16: 2. Published online 2019 Jan 5. doi: 10.1186/s12974-018-1383-2"	(75)	"Acute Ischemic Stroke" + "Neuroprotection" + "Hyperbaric oxygen therapy"	2019	8
"Federico Carbone, Aldo Bonaventura, Fabrizio Montecucco"	"Neutrophil-Related Oxidants Drive Heart and Brain Remodeling After Ischemia/Reperfusion Injury"	"Front Physiol. 2019; 10: 1587. Published online 2020 Feb 4. doi: 10.3389/fphys.2019.01587"	(68)	"Acute Ischemic Stroke" + "Neuroprotection" + "Hyperbaric oxygen therapy"	2020	8
"Jing Zeng, Long Zhu, Jing Liu, Tao Zhu, Zhaohui Xie, Xiaoou Sun, Hao Zhang"	"Metformin Protects against Oxidative Stress Injury Induced by Ischemia/Reperfusion via Regulation of the lncRNA-H19/miR-148a-3p/Rock2 Axis"	"Oxid Med Cell Longev. 2019; 2019: 8768327. Published online 2019 Dec 16. doi: 10.1155/2019/8768327"	(36)	"Acute Ischemic Stroke" + "Neuroprotection" + "Transcranial Magnetic Stimulation"	2019	8
"Jiabing Wang, Lili Huang, Chanchan Cheng, Ge Li, Jingwen Xie, Mengya Shen, Qian Chen, Wulan Li, Wenfei He, Peihong Qiu, Jianzhang Wu"	"Design, synthesis and biological evaluation of chalcone analogs with novel dual antioxidant mechanisms as potential anti-ischemic stroke agents"	"Acta Pharm Sin B. 2019 Mar; 9(2): 335–350. Published online 2019 Jan 7. doi: 10.1016/j.apsb.2019.01.003"	(35)	"Acute Ischemic Stroke" + "Neuroprotection" + "TMS"	2019	8
"Zong-Jian Liu, Yuan-Yuan Ran, Shu-Yan Qie, Wei-Jun Gong, Fu-Hai Gao, Zi-Tong Ding, Jia-Ning Xi"	"Melatonin protects against ischemic stroke by modulating microglia/macrophage polarisation toward anti-	"CNS Neurosci Ther. 2019 Dec; 25(12): 1353–1362. Published online 2019 Dec 2. doi: 10.1111/cns.13261"	(69)	"Acute Ischemic Stroke" + "Neuroprotection" + "Selective Brain Cooling"	2019	8

	inflammatory phenotype through STAT3 pathway”					
“Thomas H. Sanderson, Joseph M. Wider, Icksoo Lee, Christian A. Reynolds, Jenney Liu, Bradley Lepore, Reneé Tousignant, Melissa J. Bukowski, Hollie Johnston, Alemu Fite, Sarita Raghunayakula, John Kamholz, Lawrence I. Grossman, Karin Przyklenk, Maik Hüttemann”	“Inhibitory modulation of cytochrome c oxidase activity with specific near-infrared light wavelengths attenuates brain ischemia/reperfusion injury”	“Sci Rep. 2018; 8: 3481. Published online 2018 Feb 22. doi: 10.1038/s41598-018-21869-x”	(14)	"Acute Ischemic Stroke" + "Neuroprotection" + "Photobiomodulatio n"	2018	7
“Ahmet B. Caglayan, Mustafa C. Beker, Berrak Caglayan, Esra Yalcin, Aysun Caglayan, Burak Yulug, Lutfu Hanoglu, Selim Kutlu, Thorsten R. Doeppner, Dirk M. Hermann, Ertugrul Kilic”	“Acute and Post-acute Neuromodulation Induces Stroke Recovery by Promoting Survival Signaling, Neurogenesis, and Pyramidal Tract Plasticity”	“Front Cell Neurosci. 2019; 13: 144. Published online 2019 Apr 12. doi: 10.3389/fncel.2019.00144”	(116)	"Acute Ischemic Stroke" + "Neuroprotection" + "Transcranial Magnetic Stimulation"	2019	7
“Talia Knecht, Cesar Borlongan, Ike dela Peña”	“Combination therapy for ischemic stroke: Novel approaches to lengthen therapeutic window of tissue plasminogen activator”	“Brain Circ. 2018 Jul-Sep; 4(3): 99–108. Published online 2018 Oct 9. doi: 10.4103/bc.bc_21_18”	(15)	"Acute Ischemic Stroke" + "Neuroprotection" + "Hyperbaric oxygen therapy"	2018	7
“Minyu Zhang, Liping Xu, Hongjun Yang”	“Schisandra chinensis Fructus and Its Active Ingredients as Promising Resources for the Treatment of Neurological Diseases”	“Int J Mol Sci. 2018 Jul; 19(7): 1970. Published online 2018 Jul 6. doi: 10.3390/ijms19071970”	(47)	"Acute Ischemic Stroke" + "Neuroprotection" + "Transcranial Magnetic Stimulation"	2018	7
“Mariangela Gennaro, Alessandro Mattiello, Tommaso Pizzorusso”	“Rodent Models of Developmental Ischemic Stroke for Translational Research: Strengths and Weaknesses”	“Neural Plast. 2019; 2019: 5089321. Published online 2019 Apr 4. doi: 10.1155/2019/5089321”	(122)	"Acute Ischemic Stroke" + "Neuroprotection" + "RTMS"	2019	7
“Daniel González-Nieto, Laura Fernández-García, José Pérez-Rigueiro, Gustavo V. Guinea, Fivos Panetsos”	“Hydrogels-Assisted Cell Engraftment for Repairing the Stroke-Damaged Brain: Chimera or Reality”	“Polymers (Basel) 2018 Feb; 10(2): 184. Published online 2018 Feb 13. doi: 10.3390/polym10020184”	(78)	"Acute Ischemic Stroke" + "Neuroprotection" + "TDCS"	2018	7
“Andrea M Kuczynski, Andrew M Demchuk, Mohammed A Almekhlafi”	“Therapeutic hypothermia: Applications in adults with acute ischemic stroke”	“Brain Circ. 2019 Apr-Jun; 5(2): 43–54. Published online 2019 Jun 27. doi: 10.4103/bc.bc_5_19	(100)	"Acute Ischemic Stroke" + "Neuroprotection" + "Systemic Cooling"	2019	7
“Zhi-Gang Mei, Ya-Guang Huang, Zhi-Tao Feng, Ya-Nan Luo, Song-Bai Yang, Li-Peng Du, Kang Jiang, Xiao-Lu Liu, Xian-Yun Fu, Yi-Hui Deng, Hua-Jun Zhou	“Electroacupuncture ameliorates cerebral ischemia/reperfusion injury by suppressing autophagy via the SIRT1-FOXO1 signaling pathway”	“Aging (Albany NY) 2020 Jul 15; 12(13): 13187–13205. Published online 2020 Jul 3. doi: 10.18632/aging.103420”	(79)	"Acute Ischemic Stroke" + "Neuroprotection" + "Electro-Acupuncture"	2020	6
“Jiao Liu, Qin Wang, Shanli Yang, Jia Huang, Xiaodong Feng, Jun Peng, Zhengkun Lin, Weilin Liu, Jing Tao, Lidian Chen”	“Electroacupuncture Inhibits Apoptosis of Peri-Ischemic Regions via Modulating p38, Extracellular Signal-Regulated Kinase (ERK1/2), and c-Jun N Terminal Kinases (JNK) in Cerebral Ischemia-Reperfusion-Injured Rats”	“Med Sci Monit. 2018; 24: 4395–4404. Published online 2018 Jun 26. doi: 10.12659/MSM.908473”	(80)	"Acute Ischemic Stroke" + "Neuroprotection" + "Electro-Acupuncture"	2018	6
“Yu Zhan, Man-Zhong Li, Le Yang, Xue-Feng Feng, Qiu-Xia Zhang, Nan Zhang, Yuan-Yuan Zhao, Hui Zhao”	“An MRI Study of Neurovascular Restorative After Combination Treatment With Xiaoshuan Enteric-Coated Capsule and Enriched Environment in Rats After Stroke”	“Front Neurosci. 2019; 13: 701. Published online 2019 Jul 9. doi: 10.3389/fnins.2019.00701”	(6)	"Acute Ischemic Stroke" + "Neuroprotection" + "Rehabilitative Intervention"	2019	6
“Fan Su, Wendong Xu”	“Enhancing Brain Plasticity to Promote Stroke Recovery”	“Front Neurol. 2020; 11: 554089. Published online 2020 Oct 30. doi: 10.3389/fneur.2020.554089”	(109)	"Acute Ischemic Stroke" + "Neuroprotection" + "TDCS"	2020	6
“Annette Gower, Mario Tiberi”	“The Intersection of Central Dopamine System and Stroke: Potential Avenues Aiming at Enhancement of Motor Recovery”	“Front Synaptic Neurosci. 2018; 10: 18. Published online 2018 Jul 6. doi: 10.3389/fnsyn.2018.00018”	(32)	"Acute Ischemic Stroke" + "Neuroprotection" + "TDCS"	2018	6

"Ying Xing, Min Zhang, Wen-Bin Li, Fang Dong, Feng Zhang	"Mechanisms Involved in the Neuroprotection of Electroacupuncture Therapy for Ischemic Stroke"	"Front Neurosci. 2018; 12: 929. Published online 2018 Dec 11. doi: 10.3389/fnins.2018.00929	(23)	"Acute Ischemic Stroke" + "Neuroprotection" + "Electro-Acupuncture"	2018	6
Rong Sha, Bo Zhang, Xiaohua Han, Jiaojiao Peng, Caixia Zheng, Fengxia Zhang, Xiaolin Huang"	"Electroacupuncture Alleviates Ischemic Brain Injury by Inhibiting the miR-223/NLRP3 Pathway"	"Med Sci Monit. 2019; 25: 4723–4733. Published online 2019 Jun 25. doi: 10.12659/MSM.917213"	(70)	"Acute Ischemic Stroke" + "Neuroprotection" + "Electro-Acupuncture"	2019	6
"Bei-Yao Gao, Cheng-Cheng Sun, Guo-Hua Xia, Shao-Ting Zhou, Ye Zhang, Ye-Ran Mao, Pei-Le Liu, Ya Zheng, Dan Zhao, Xu-Tong Li, Janie Xu, Dong-Sheng Xu, Yu-Long Bai"	"Paired associated magnetic stimulation promotes neural repair in the rat middle cerebral artery occlusion model of stroke"	"Neural Regen Res. 2020 Nov; 15(11): 2047–2056. Published online 2020 May 11. doi: 10.4103/1673-5374.282266"	(38)	"Acute Ischemic Stroke" + "Neuroprotection" + "Electro-Acupuncture"	2020	6
"Zheng Zhang, Linlei Zhang, Yuchuan Ding, Zhao Han, Xunming Ji"	"Effects of Therapeutic Hypothermia Combined with Other Neuroprotective Strategies on Ischemic Stroke: Review of Evidence"	"Aging Dis. 2018 Jun; 9(3): 507–522. Published online 2018 Jun 1. doi: 10.14336/AD.2017.0628"	(101)	"Acute Ischemic Stroke" + "Neuroprotection" + "Hyperbaric oxygen therapy"	2018	6
"Christian Huber, Mitchell Huber, Yuchuan Ding"	"Evidence and opportunities of hypothermia in acute ischemic stroke: Clinical trials of systemic versus selective hypothermia"	"Brain Circ. 2019 Oct-Dec; 5(4): 195–202. Published online 2019 Dec 27. doi: 10.4103/bc.bc_25_19"	(61)	"Acute Ischemic Stroke" + "Neuroprotection" + "Systemic Cooling"	2019	6
"Thomas K. Mattingly, Stephen P. Lownie"	"Cold blood perfusion for selective hypothermia in acute ischemic stroke"	"Brain Circ. 2019 Oct-Dec; 5(4): 187–194. Published online 2019 Dec 27. doi: 10.4103/bc.bc_17_19"	(93)	"Acute Ischemic Stroke" + "Neuroprotection" + "Systemic Cooling"	2019	6
"Ya-Nan Tang, Gao-Feng Zhang, Huai-Long Chen, Xiao-Peng Sun, Wei-Wei Qin, Fei Shi, Li-Xin Sun, Xiao-Na Xu, Ming-Shan Wang"	"Selective brain hypothermia-induced neuroprotection against focal cerebral ischemia/reperfusion injury is associated with Fis1 inhibition"	"Neural Regen Res. 2020 May; 15(5): 903–911. Published online 2019 Nov 8. doi: 10.4103/1673-5374.268973"	(94)	"Acute Ischemic Stroke" + "Neuroprotection" + "Selective Brain Cooling"	2020	6
"Cheng-Ting Jiang, Wan-Feng Wu, Yi-Hui Deng, Jin-Wen Ge"	"Modulators of microglia activation and polarisation in ischemic stroke"	"Mol Med Rep. 2020 May; 21(5): 2006–2018. Published online 2020 Feb 26. doi: 10.3892/mmr.2020.11003"	(24)	"Acute Ischemic Stroke" + "Neuroprotection" + "Photobiomodulation"	2020	5
"Li-Ru Zhao, Alison Willing"	"Enhancing endogenous capacity to repair a stroke-damaged brain: An evolving field for stroke research"	"Prog Neurobiol. Author manuscript; available in PMC 2018 Aug 3. Published in final edited form as: Prog Neurobiol. 2018 Apr-May; 163-164: 5–26. Published online 2018 Feb 21. doi: 10.1016/j.pneurobio.2018.01.004"	(119)	"Acute Ischemic Stroke" + "Neuroprotection" + "TDCS"	2018	5
"Reggie H. C. Lee, Michelle H. H. Lee, Celeste Y. C. Wu, Alexandre Couto e Silva, Harlee E. Possoit, Tsung-Han Hsieh, Alireza Minagar, Hung Wen Lin"	"Cerebral ischemia and neuroregeneration"	"Neural Regen Res. 2018 Mar; 13(3): 373–385. doi: 10.4103/1673-5374.228711"	(81)	"Acute Ischemic Stroke" + "Neuroprotection" + "TDCS"	2018	5
"Wei Liu, Xiaohui Wang, Margaret O'Connor, Guan Wang, Fang Han"	"Brain-Derived Neurotrophic Factor and Its Potential Therapeutic Role in Stroke Comorbidities"	"Neural Plast. 2020; 2020: 1969482. Published online 2020 Jan 27. doi: 10.1155/2020/1969482"	(12)	"Acute Ischemic Stroke" + "Neuroprotection" + "TDCS"	2020	5
"Ruiqiao Guan, Wei Zou, Xiaohong Dai, Xueping Yu, Hao Liu, Qiuxin Chen, Wei Teng"	"Mitophagy, a potential therapeutic target for stroke"	"J Biomed Sci. 2018; 25: 87. Published online 2018 Nov 30. doi: 10.1186/s12929-018-0487-4"	(34)	"Acute Ischemic Stroke" + "Neuroprotection" + "Electro-Acupuncture"	2018	5
"Xingping Qin, Jing Cheng, Yi Zhong, Omer Kamal Mahgoub, Farhana Akter, Yanqin Fan, Mohammed Aldughaim, Qiurong Xie, Lingxia Qin, Lijuan Gu, Zhihong Jian, Xiaoxing Xiong, Renzhong Liu"	"Mechanism and Treatment Related to Oxidative Stress in Neonatal Hypoxic-Ischemic Encephalopathy"	"Front Mol Neurosci. 2019; 12: 88. Published online 2019 Apr 11. doi: 10.3389/fnmol.2019.00088"	(13)	"Acute Ischemic Stroke" + "Neuroprotection" + "Hyperbaric oxygen therapy"	2019	5
"Ricardo O. S. Soares, Daniele M. Losada, Maria	"Ischemia/Reperfusion Injury Revisited: An Overview of the	"Int J Mol Sci. 2019 Oct; 20(20): 5034. Published online	(10)	"Acute Ischemic Stroke" + "Neuroprotection" +	2019	5

C. Jordani, Paulo Évora, Orlando Castro-e-Silva”	Latest Pharmacological Strategies”	2019 Oct 11. doi: 10.3390/ijms20205034”		"Hyperbaric oxygen therapy"		
"Maliheh Mohamadpour, Kristen Whitney, Peter J. Bergold”	"The Importance of Therapeutic Time Window in the Treatment of Traumatic Brain Injury”	"Front Neurosci. 2019; 13: 07. Published online 2019 Jan 23. doi: 10.3389/fnins.2019.00007”	(19)	"Acute Ischemic Stroke" + "Neuroprotection" + "Hyperbaric oxygen therapy"	2018	5
"Megan Finch-Edmondson, Catherine Morgan, Rod W. Hunt, Iona Novak	"Emergent Prophylactic, Reparative and Restorative Brain Interventions for Infants Born Preterm With Cerebral Palsy”	"Front Physiol. 2019; 10: 15. Published online 2019 Jan 28. doi: 10.3389/fphys.2019.00015	(59)	"Acute Ischemic Stroke" + "Neuroprotection" + "Transcranial Magnetic Stimulation"	2019	5
"Steven C. Cramer”	"Treatments to Promote Neural Repair after Stroke”	"J Stroke. 2018 Jan; 20(1): 57–70. Published online 2018 Jan 31. doi: 10.5853/jos.2017.02796”	(62)	"Acute Ischemic Stroke" + "Neuroprotection" + "Transcranial Magnetic Stimulation"	2018	5
"Aravind S. Kshatri, Alberto Gonzalez-Hernandez, Teresa Giraldez”	"Physiological Roles and Therapeutic Potential of Ca2+ Activated Potassium Channels in the Nervous System”	"Front Mol Neurosci. 2018; 11: 258. Published online 2018 Jul 30. doi: 10.3389/fnmol.2018.00258 ”	(29)	"Acute Ischemic Stroke" + "Neuroprotection" + "Transcranial Magnetic Stimulation"	2018	5
"Marta Wolska, Joanna Jarosz-Popek, Eva Junger, Zofia Wicik, Tahmina Porshoor, Lucia Sharif, Pamela Czajka, Marek Postula, Dagmara Mirowska-Guzel, Anna Czlonkowska, Ceren Eyileten”	"Long Non-coding RNAs as Promising Therapeutic Approach in Ischemic Stroke: a Comprehensive Review”	"Mol Neurobiol. 2021; 58(4): 1664–1682. Published online 2020 Nov 24. doi: 10.1007/s12035-020-02206-8”	(121)	"Acute Ischemic Stroke" + "Neuroprotection" + "RTMS"	2020	5
"Ceren Eyileten, Lucia Sharif, Zofia Wicik, Daniel Jakubik, Joanna Jarosz-Popek, Aleksandra Soplinska, Marek Postula, Anna Czlonkowska, Agnieszka Kaplon-Cieslicka, Dagmara Mirowska-Guzel”	"The Relation of the Brain-Derived Neurotrophic Factor with MicroRNAs in Neurodegenerative Diseases and Ischemic Stroke”	"Mol Neurobiol. 2021; 58(1): 329–347. Published online 2020 Sep 17. doi: 10.1007/s12035-020-02101-2”	(57)	"Acute Ischemic Stroke" + "Neuroprotection" + "RTMS"	2020	5
"Travis C. Jackson, Patrick M. Kochanek”	"A New Vision for Therapeutic Hypothermia in the Era of Targeted Temperature Management: A Speculative Synthesis”	"Ther Hypothermia Temp Manag. 2019 Mar 1; 9(1): 13–47. Published online 2019 Mar 6. doi: 10.1089/ther.2019.0001”	(99)	"Acute Ischemic Stroke" + "Neuroprotection" + "Systemic Cooling"	2019	5
"Clémence DISDIER, Barbara S STONESTREET”	"Hypoxic-ischemic related cerebrovascular changes and potential therapeutic strategies in the neonatal brain”	"J Neurosci Res. Author manuscript; available in PMC 2021 Jan 1. Published in final edited form as: J Neurosci Res. 2020 Jul; 98(7): 1468–1484. Published online 2020 Feb 14. doi: 10.1002/jnr.24590”	(52)	"Acute Ischemic Stroke" + "Neuroprotection" + "Selective Brain Cooling"	2020	5
"Ming-Shuo Sun, Hang Jin, Xin Sun, Shuo Huang, Fu-Liang Zhang, Zhen-Ni Guo, Yi Yang”	"Free Radical Damage in Ischemia-Reperfusion Injury: An Obstacle in Acute Ischemic Stroke after Revascularization Therapy”	"Oxid Med Cell Longev. 2018; 2018: 3804979. Published online 2018 Jan 31. doi: 10.1155/2018/3804979”	(31)	"Acute Ischemic Stroke" + "Neuroprotection" + "Selective Brain Hypothermia"	2018	5
"Man Li, Jing Liu, Ying Bi, Jixiang Chen, Lei Zhao”	"Potential Medications or Compounds Acting on Toll-like Receptors in Cerebral Ischemia”	"Curr Neuropharmacol. 2018 Feb; 16(2): 160–175. Published online 2018 Feb. doi: 10.2174/1570159X15666170601125139”	(27)	"Acute Ischemic Stroke" + "Neuroprotection" + "Electro-Acupuncture"	2018	4
"Abir A. Rahman, Narayanappa Amruta, Emmanuel Pinteaux, Gregory J. Bix”	"Neurogenesis After Stroke: A Therapeutic Perspective”	"Transl Stroke Res. 2021; 12(1): 1–14. Published online 2020 Aug 29. doi: 10.1007/s12975-020-00841-w”	(58)	"Acute Ischemic Stroke" + "Neuroprotection" + "Photobiomodulation"	2020	4
"Manisha Singh, Pranav K. Pandey, Ashu Bhasin, M. V. Padma, Sujata Mohanty”	"Application of Stem Cells in Stroke: A Multifactorial Approach”	"Front Neurosci. 2020; 14: 473. Published online 2020 Jun 9. doi: 10.3389/fnins.2020.00473”	(1)	"Acute Ischemic Stroke" + "Neuroprotection" + "TDCS"	2020	4
"Xiaoqin Duan, Gang Yao, Zhongliang Liu, Ranji Cui, Wei Yang”	"Mechanisms of Transcranial Magnetic Stimulation Treating on Post-stroke Depression”	"Front Hum Neurosci. 2018; 12: 215. Published online 2018 May 30. doi: 10.3389/fnhum.2018.00215”	(56)	"Acute Ischemic Stroke" + "Neuroprotection" + "TDCS"	2018	4

"Elliot H. Choi, Agata Blasiak, Joonho Lee, In Hong Yang"	"Modulation of Neural Activity for Myelination in the Central Nervous System"	"Front Neurosci. 2019; 13: 952. Published online 2019 Sep 6. doi: 10.3389/fnins.2019.00952"	(114)	"Acute Ischemic Stroke" + "Neuroprotection" + "Transcranial Magnetic Stimulation"	2019	4
"Ramón Iglesias-Rey, José Castillo"	"New strategies for ischemic stroke: internal photobiomodulation therapy"	"Neural Regen Res. 2020 Sep; 15(9): 1658–1659. Published online 2020 Feb 28. doi: 10.4103/1673-5374.276328"	(105)	"Acute Ischemic Stroke" + "Neuroprotection" + "Photobiomodulation"	2020	4
"Iris Escobar, Jing Xu, Charles W. Jackson, Miguel A. Perez-Pinzon"	"Altered Neural Networks in the Papez Circuit: Implications for Cognitive Dysfunction after Cerebral Ischemia"	"J Alzheimers Dis. 2019; 67(2): 425–446. Pre-published online 2018 Dec 20. Published online 2019 Jan 22. doi: 10.3233/JAD-180875"	(60)	"Acute Ischemic Stroke" + "Neuroprotection" + "TDCS"	2018	4
